# Hijacking host extracellular vesicle machinery by hepatotropic viruses: current understandings and future prospects

**DOI:** 10.1186/s12929-024-01063-0

**Published:** 2024-10-05

**Authors:** Yu-De Chu, Mi-Chi Chen, Chau-Ting Yeh, Ming-Wei Lai

**Affiliations:** 1 Liver Research Center, Chang Gung Memorial Hospital, 5F., No. 15, Wenhua 1st Rd., Guishan Dist., Taoyuan City, 333 Taiwan; 2 Department of Pediatric, Chang Gung Memorial Hospital, Taoyuan, Taiwan; 3 Institute of Stem Cell and Translational Cancer Research, Chang Gung Memorial Hospital, Taoyuan, Taiwan

**Keywords:** Extracellular vesicle subtypes, Hepatitis viruses, Exosomes, Microvesicles, Large oncosomes, ESCRT, Ceramide

## Abstract

Recent advances in studies exploring the roles of extracellular vesicles (EVs) in viral transmission and replication have illuminated hepatotropic viruses, such as hepatitis A (HAV), hepatitis B (HBV), hepatitis C (HCV), hepatitis D (HDV), and hepatitis E (HEV). While previous investigations have uncovered these viruses’ ability to exploit cellular EV pathways for replication and transmission, most have focused on the impacts of exosomal pathways. With an improved understanding of EVs, four main subtypes, including exosomes, microvesicles, large oncosomes, and apoptotic bodies, have been categorized based on size and biogenic pathways. However, there remains a noticeable gap in comprehensive reviews summarizing recent findings and outlining future perspectives for EV studies related to hepatotropic viruses. This review aims to consolidate insights into EV pathways utilized by hepatotropic viruses, offering guidance for the future research direction in this field. By comprehending the diverse range of hepatotropic virus-associated EVs and their role in cellular communication during productive viral infections, this review may offer valuable insights for targeting therapeutics and devising strategies to combat virulent hepatotropic virus infections and the associated incidence of liver cancer.

## Introduction

In the 1980s, scientific exploration of extracellular vesicles (EVs) began, initially focusing on reticulocyte maturation, which revealed EVs containing transferrin receptors were crucial for modulating reticulocyte maturation [1]. Subsequently, these EVs were identified as “exosomes”, originating from fusion of multivesicular bodies (MVBs), displaying phospholipid-bilayer-enclosed spherical structures and carrying diverse cargos [2, 3].

Current understanding of EVs encompasses various subtypes released into body fluids like blood, urine, and cerebrospinal fluid, serving as rich reservoirs. These subtypes include exosomes (40–100 nm), microvesicles (MVs, 100–1000 nm), apoptotic bodies (ApoBDs, 50–500 nm), and large oncosomes (1–10 μm) [4]. These particles mediate cell-to-cell communication, delivering functional biomolecules critical for physiological functions and influencing recipient cells’ composition and function [5]. They also serve as markers for diagnosis, disease progression, and therapeutic targeting, especially in liver diseases [6, 7].

Analyzing EVs poses challenges due to their size and heterogeneity [8, 9]. Specific methodologies and functional/physical analyses like electron microscopy (EM) and nanoparticle tracking analysis (NTA) are recommended. Size exclusion chromatography and ultracentrifugation remain vital tools in EV research.

Recent research on EVs has uncovered the exploitation of cellular EV pathways by viruses, including Herpesviruses [10], as well as hepatotropic viruses [11–13], for replication and transmission. However, a gap exists in comprehensive reviews summarizing these latest findings and outlining future perspectives. This review aims to consolidate insights into EV pathways used by hepatotropic viruses, prospecting future research.

### Diverse biogenesis of EV subtypes

**Fig. 1:**
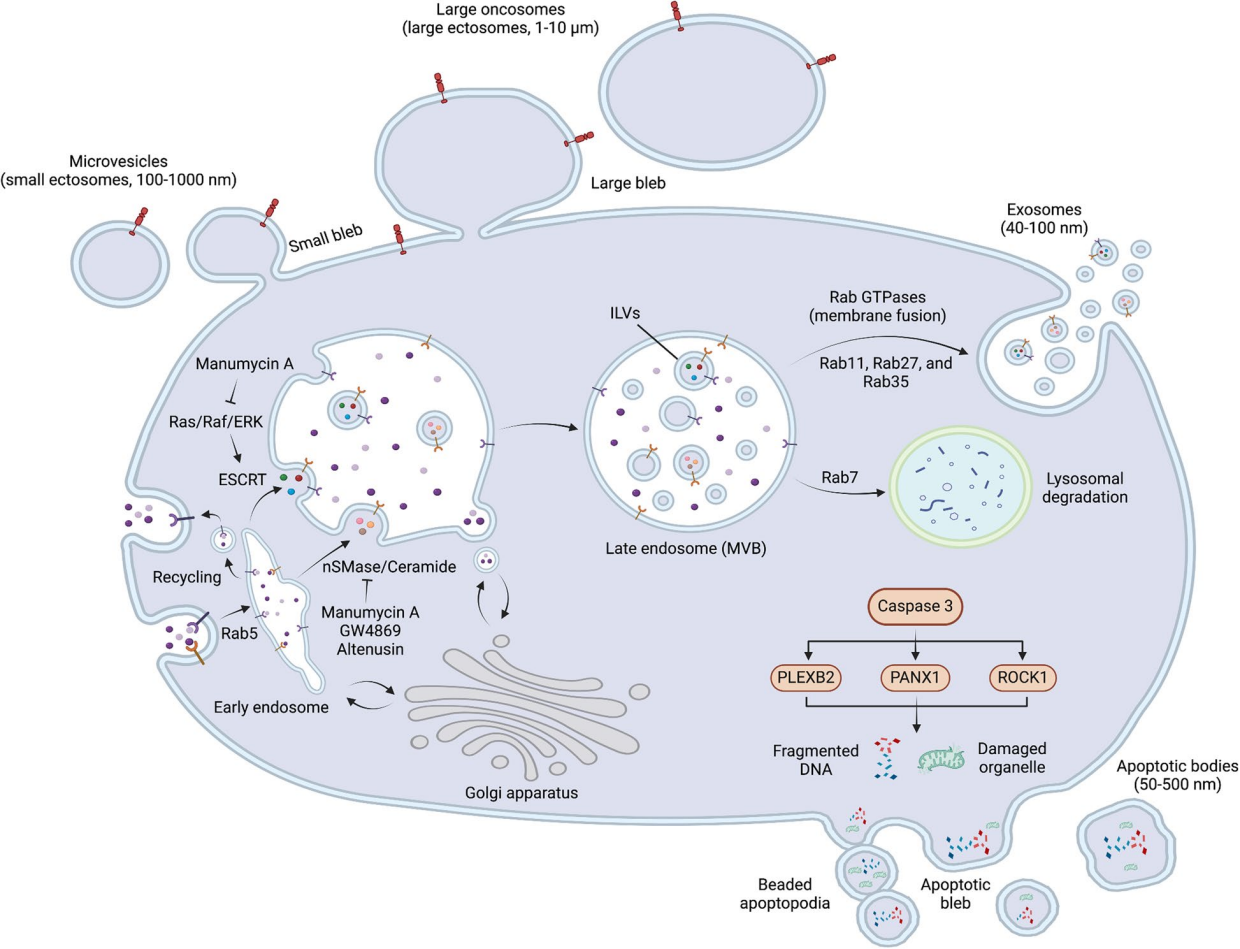
Biogenesis pathways of main extracellular vesicles. The four primary types of extracellular vesicles, exosomes (40–100 nm), microvesicles (100–1000 nm), large oncosomes (1–10 μm), and apoptotic bodies (50–500 nm), are illustrated. The initiation step of exosome formation involves the creation of early endosomes, which happens subsequent to the endocytosis or uptake of extracellular fluids, particles, and viruses through receptor- and Clathrin-dependent or independent routes. Notably, if there are any plasma membrane receptors or membrane-integrated proteins located within the region of the endocytic membrane, their orientation changes from facing the outside of the cells to facing the endosomal lumen after endocytosis-mediated internalization. These receptors can then either be recycled to the plasma membrane or retained within the endosome. Exosomes are then formed through endosomal membrane inward budding to generate intraluminal vesicles (ILVs) via endosomal sorting complexes required for transport (ESCRT) complexes or an ESCRT-independent route through lipid rafts, such as the membrane-associated neutral sphingomyelinase (nSMase) and the ceramide-triggered pathway. As ILVs form, the orientation of plasma membrane receptors or membrane-integrated proteins within the endosome undergoes another change, transitioning from facing the endosomal lumen to facing the outside of the ILV. Both ESCRT-dependent and nSMase/ceramide-triggered exosomes can be inhibited by blockers such as Manumycin A, GW4869, and Altenusin. As ILVs accumulate within a single endosome during endosome maturation, the early endosome progresses into the late endosome, also referred to as the multivesicular bodies (MVBs). MVBs can proceed through two pathways: fusion with lysosomes for degradation, which involves the small GTPase Rab7, or secretion into the extracellular space as exosomes after MVB-plasma membrane fusion, a process regulated by small GTPases such as Rab11, Rab27 and Rab35. Microvesicles and large oncosomes are categorized as ectosomes, originating as outward buds from the plasma membrane. Apoptotic bodies result from the orderly fragmentation of apoptotic cells, and the formation of apoptotic bodies involves key roles played by caspase-3 substrates, including ROCK1, PANX1, and PLEXB2

EVs are classified based on distinct biogenesis pathways [4] (Fig. 1). Four main types, including exosomes, MVs, large oncosomes, and ApoBDs, are identified. Among these, MVs and large oncosomes belong to the category of ectosomes. The formation of exosomes and ectosomes relies on local microdomains assembled in endocytic membranes for exosomes and in the plasma membrane for ectosomes [14]. These microdomains govern the accumulation of proteins and RNAs associated with their cytosolic surface, leading to membrane budding inward for exosome precursors, known as intraluminal vesicles (ILVs), and outward for ectosomes. These two types of vesicles differ in size, with exosomes typically ranging from 40 to 100 nm and ectosomes ranging from 100 to 10,000 nm, as well as in the mechanisms of assembly, composition, and regulation of release, although there are some partially overlapped mechanisms [14]. Further details of these vesicles are described in the section below.

### Exosome biogenesis

Exosomes, the first discovered EV subtype [1], undergo a series of steps during biogenesis. The initiation step involves the formation of early endosomes, which occurs after the endocytosis or uptake of extracellular fluids, particles, and viruses through receptor- and Clathrin-dependent or independent routes [15]. Importantly, if there are any plasma membrane receptors or membrane-integrated proteins located within the region of the endocytic membrane, their orientation changes from facing the outside of the cells to facing the endosomal lumen after endocytosis-mediated internalization (Fig. 1). These receptors can then either be recycled to the plasma membrane or retained within the endosome. Subsequently, ILVs are formed through inward budding of the endosomal membrane [16]. As ILVs form, the orientation of plasma membrane receptors or membrane-integrated proteins within the endosome undergoes another change, transitioning from facing the endosomal lumen to facing the outside of the ILV (Fig. 1).

The process of ILV formation is facilitated by endosomal sorting complexes required for transport (ESCRT) complexes (ESCRT-0, ESCRT-I, ESCRT-II, and ESCRT-III) and the ALG-interacting protein X (ALIX)-Syntenin complex (ESCRT-dependent pathway) [16]. In this pathway, ESCRT-0, along with disassembly and deubiquitinating enzymes, as well as the ESCRT accessory protein Vacuolar Protein Sorting 4 (VPS4), cluster on the endosomal membrane at the cytoplasmic side to sort cargos [17]. The Hepatocyte Growth Factor-Regulated Tyrosine Kinase Substrate (HRS) subunit of ESCRT-0 coordinates early steps in ILV biogenesis by binding to cargoes and recruiting clathrin to the early endosome [18]. Subsequently, ESCRT-I, ESCRT-II, and ESCRT-III are sequentially recruited to maturing endosomes [17]. ESCRT-II induces the formation of ESCRT-III filaments, which sever the nascent ILVs from the endosome membrane [19]. Alternatively, the ESCRT-III complex can be recruited by ALIX, binding to lysobisphosphatidic acid on the endosomal membrane [20]. ESCRT-III may sense negative membrane curvature or promote membrane bending to drive fission [21, 22]. Notably, studies have shown that silencing ESCRT-0 proteins HRS or Signal Transducing Adaptor Molecule 1 (STAM1), or ESCRT-I subunit Tumor Susceptibility 101 (TSG101), reduces small EV secretion, suggesting redundancy in ESCRT-II and ESCRT-III components [23]. Variations on the ESCRT pathway, such as the syndecan–syntenin–ALIX pathway, are specifically compromised by knockdown of genes encoding ESCRT-I protein TSG101, ESCRT-II subunit Vacuolar Protein Sorting 22 (VPS22), or ESCRT-III filament protein Charged Multivesicular Body Protein 4A (CHMP4) [24]. Syndecan-1 interacts with syntenin and ALIX on endosomes, thereby facilitating cargo sorting [24–26]. Thus, syndecan-1, syntenin, and ALIX play roles in cargo sorting during this process.

Currently, it is recognized that ILV production can also occur via ESCRT-independent processes involving lipid rafts [27, 28]. These lipid rafts, rich in cholesterol and sphingolipids, are subject to the activity of the neutral sphingomyelinase (nSMase) family, membrane-bound enzymes that convert sphingolipids, specifically sphingomyelin, to ceramide, a cone-shaped rigid lipid [29, 30]. Once converted, ceramide can form lipid raft microdomains, such as ceramide-enriched membrane domains, and initiate the formation and inward budding of ILVs into the endosome. Due to their unique cone-shaped structure, ceramides induce spontaneous membrane invagination, facilitating ILV formation within endosomes and maintaining vesicle shape and structure [29–31].

Both ESCRT-dependent and nSMase/ceramide-triggered exosomes can be inhibited by blockers such as Manumycin A, GW4869 and Altenusin [32]. Manumycin A functions as an inhibitor for both ESCRT and nSMase/ceramide-dependent pathways [33, 34]. In the ESCRT-dependent pathway, Manumycin A targets ESCRT-0 proteins HRS, ALIX, and Rab27a, leading to the inhibition of exosome biogenesis and secretion. This inhibition primarily occurs through targeted suppression of the Ras/Raf/ERK1/2 signaling pathway [34]. Additionally, Manumycin A irreversibly inhibits nSMase, further reducing exosome biogenesis and secretion [33]. GW4869 selectively inhibits nSMase2, while Altenusin selectively inhibits a broad range of nSMases [35, 36]. These inhibitors serve as valuable tools for investigating the physiological and pathological roles of exosomes.

As ILVs accumulate within a single endosome during endosome maturation, the early endosome progresses into the late endosome, also referred to as the MVB [37, 38]. MVBs can proceed through two pathways: fusion with lysosomes for degradation, which involves the small GTPase Rab7 [39], or secretion into the extracellular space as exosomes following fusion of the MVB with the plasma membrane, a process regulated by small GTPases such as Rab11, Rab27 and Rab35 [37, 38].

### Ectosome biogenesis

MVs and large oncosomes fall under ectosomes, distinct from exosomes, originating as outward buds from the plasma membrane. Small ectosomes like MVs share machinery with exosomes, involving tetraspanin proteins, such as CD9, CD63, and CD81, which interacts with Ezrin, Radixin, Moesin (ERM) and Glu-Trp-Ile EWI Motif-Containing Protein (EWI) proteins connecting to the actin cytoskeleton and impact plasma membrane organization, signaling, cargo sorting, and vesicle budding [40]. For example, CD82 recruits the ERM protein ezrin to membrane blebs for release in ectosomes [41–43]. Protrusions like filopodia, cilia, and microvilli promote MV shedding after the formation of ectosomal blebs [44–46], e.g., Human immunodeficiency virus 1 (HIV-1) particles assembling at filopodia tips, suggesting potential contributions to both retrovirion and ectosome biogenesis [47].

Large ectosome formation, on the other hand, is less understood than exosomes and small ectosomes. It remains uncertain whether early ESCRT machinery or features of exosome or small ectosome biogenesis are involved. Actin cytoskeleton rearrangements underlie plasma membrane blebbing and scission to release large EVs, with molecular reorganizations and alterations in proteins, lipids, and electrolyte levels implicated in the process [48]. Local disassembly of the cortical actin cytoskeleton, combined with actomyosin contractility, can promote plasma membrane blebbing and the subsequent formation of large ectosomes, especially in non-apoptotic cancer cells transitioning to a more migratory and metastatic phenotype [49].

Both large and small EVs contain the lipid raft marker caveolin-1 (CAV1) [50], suggesting an association with or derivation from lipid raft-associated membrane domains. CAV1, known for regulating small EV biogenesis through cholesterol binding [51], may apply to ectosomes. However, systematic studies on cholesterol and lipid rafts in EV biogenesis are lacking, leaving the possibility that the ectosomal membrane is derived from the plasma membrane and associated lipid rafts.

### Apoptotic bodies (ApoBDs) biogenesis

The biogenesis of ApoBDs differs significantly from other EV subtypes, arising from the ordered fragmentation of apoptotic cells during programmed cell death. This process progresses through several stages, including nuclear chromatin condensation, nuclear splitting, micronuclei appearance, membrane blebbing, and cellular content splitting into ApoBDs [52, 53]. Caspase-3 substrates, including Rho Associated Coiled-Coil Containing Protein Kinase 1 (ROCK1), Pannexin 1 (PANX1), and Plexin B2 (PLEXB2), play key roles in the formation of ApoBDs [52, 53]. ApoBDs can also form from protrusions known as apoptopodia [52, 53]. For instance, ROCK1 activates actomyosin contractility, leading to blebbing either directly from the plasma membrane or from the tips of surface protrusions called apoptopodia [52, 53]. Existing data suggest that membrane blebbing is, at least in part, mediated by actin-myosin interaction [54, 55]. In normal development, most ApoBDs are phagocytosed by macrophages and cleared locally [56]. However, it has been reported that the process of apoptotic cell disassembly and the removal of apoptotic material by phagocytes are rapid, limiting the presence of ApoBDs in vivo [57, 58].

### EVs mediate the transmission of hepatotropic viruses

A multitude of hepatotropic viruses has been identified and extensively scrutinized for their role in precipitating liver diseases, encompassing both acute and chronic hepatitis, as well as hepatocellular carcinoma (HCC) [59]. Currently, hepatotropic virus infections persist as a formidable public health challenge, contributing significantly to morbidity and an annual global mortality of approximately 1.5 million deaths [60]. Five prevalent viruses, namely hepatitis A (HAV), hepatitis B (HBV), hepatitis C (HCV), hepatitis D (HDV), and hepatitis E (HEV), emerge as major contributors to various liver diseases, each displaying distinct geographical localizations [61]. Notably, HBV and HCV impose the most substantial socioeconomic burdens, particularly in developing regions like Africa and Asia [61]. These viruses adeptly co-opt host materials for replication, establishing prolonged persistence through varying strategies. Their life cycle commences with attachment and entry into hepatocytes, relying on unique cell surface receptors, such as sodium taurocholate cotransporting polypeptide (NTCP) for HBV and CD81 for HCV [62, 63]. Subsequently, hepatotropic viruses manipulate host transcriptional machinery and cellular resources for replication, leading to uncontrolled viral proliferation, massive hepatocyte necrosis, inflammatory infiltration, and the onset of severe conditions like cirrhosis, HCC, or other critical illnesses [59].

Growing evidence indicates that EVs can function as carriers for these viruses, directly contributing to viral replication, transmission, or pathogenesis [64, 65]. The involvement of ESCRT components in viral capsid packaging and the maturation of enveloped viruses represents the initial evidence supporting this idea [66]. Recent studies have further illuminated the presence of viral components within EVs, particularly in exosomes and MVs, revealing that hepatotropic viruses exploit EVs to replicate, transmit their genome, and establish persistent infections. These viruses can conceal within EVs through ESCRT-dependent or independent viral budding modes, evading immune detection [67, 68].

### Canonical HAV life cycle

**Fig. 2:**
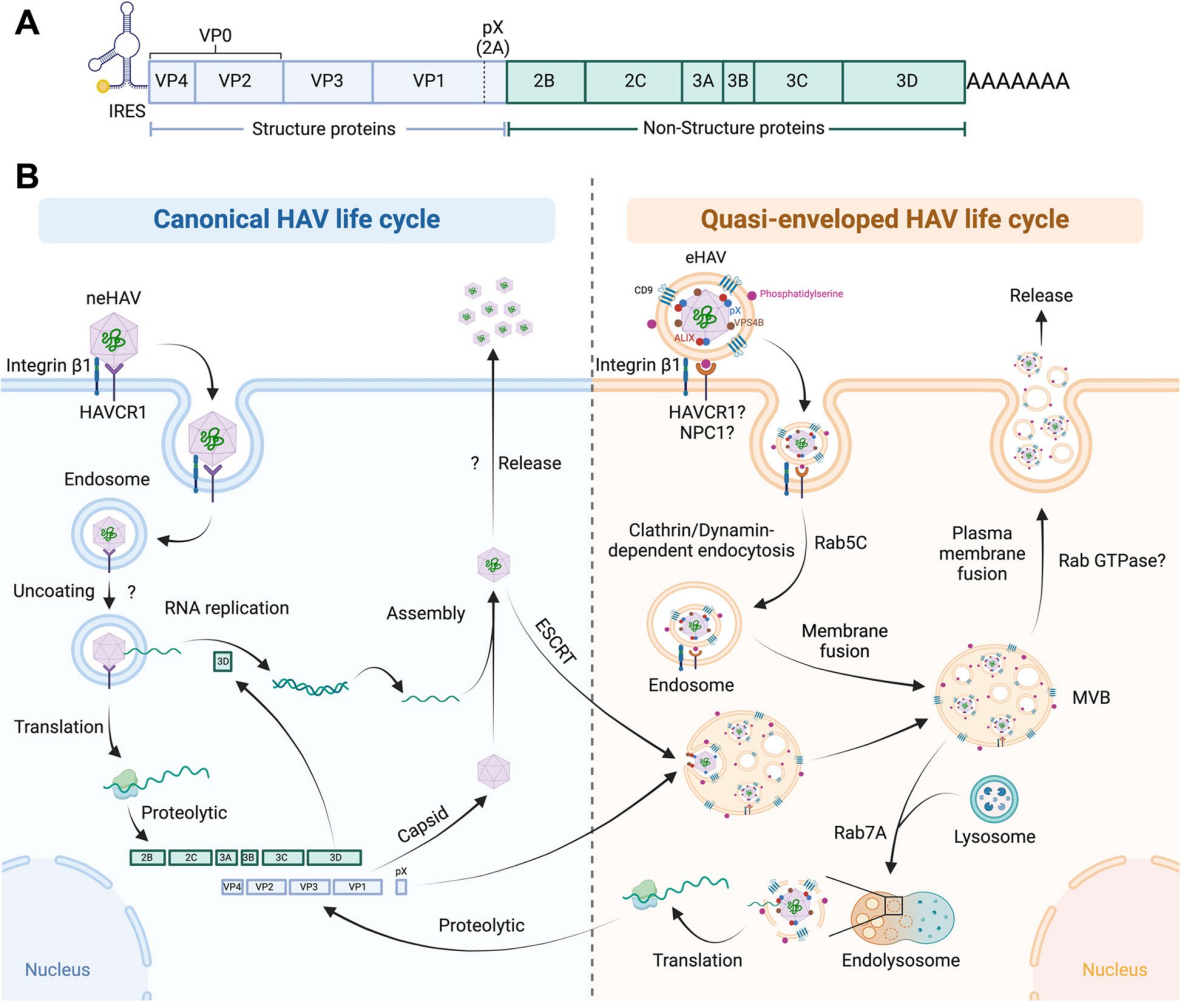
The crosstalk of canonical and extracellular vesicle-mediated HAV life cycle. **A** The schematic illustrates the genome structure of HAV, consisting of four capsid proteins (VP1, VP2, VP3, VP4) and seven nonstructural polypeptides (2A, 2B, 2C, 3A, 3B, 3C, 3D). Note that 2A is functionally equivalent to pX. **B** Non-enveloped HAV (neHAV) enters host cells by binding to receptors such as HAV cellular receptor 1 (HAVCR1) and Integrin β1, followed by endocytosis. Although the mechanisms and players involved in this process are currently unknown, upon uncoating, HAV RNA is released into the cytoplasm and utilizes host translation machinery to produce polyprotein. After proteolytic processing, the product of 3D (RNA-dependent RNA polymerase) is employed to replicate the HAV genome, which is then assembled into pre-assembled capsids and released. The mechanisms of neHAV release remain unclear. For quasi-enveloped exosomal HAV (eHAV), it is currently considered that following HAVCR1 binding to phosphatidylserine, Clathrin/dynamin-dependent endocytosis, facilitated by integrin β1, coordinates the entry of eHAV into cells and forms endosome, regulated by Rab5C. The endocytic endosome can then fuse with other endosomes or undergo maturation to form multivesicular bodies (MVBs), which can further fuse with lysosomes, facilitated by Rab7A, to release the eHAV genome and facilitate replication, or fuse with the plasma membrane to release eHAV as exosomes, although the Rabs participating in this process remain unknown. The current understanding of eHAV biogenesis from packaged neHAV involves host proteins associated with the ESCRT, specifically VPS4B and ALIX. The viral protein pX, present on the surface of eHAV but absent in non-enveloped virions, plays a crucial role in its biogenesis, potentially through interaction with ALIX via the C-terminal portion of pX, facilitating the conversion of neHAV into intraluminal vesicles (ILVs) within an MVB. Subsequently, these ILVs are secreted as eHAV after fusion of the MVB with the plasma membrane. The question mark denotes an unknown or unclear process and molecular mechanism

HAV, a small RNA virus classified within the *Picornaviridae* family, exhibits typical features characteristic of a classic non-enveloped virus. With a 7500-nucleotide positive-strand RNA genome and a diameter ranging from 27 to 32 nm, it encodes a sizable polyprotein. Upon binding to receptors such as HAV cellular receptor 1 (HAVCR1) with assistance from integrin β1, the virion enters cells [69]. Translation is initiated upon uncoating and is regulated by an internal ribosome entrance site (IRES) in the cytoplasm [70] (Fig. 2A). For translation initiation, HAV relies on intact eukaryotic initiation factor 4G (eIF4G) [71]. The translated polyprotein then undergoes proteolysis, resulting in four capsid proteins (VP1, VP2, VP3, VP4) and seven nonstructural polypeptides (2A, 2B, 2C, 3A, 3B, 3C, 3D) [72]. Notably, 2A is subsequently considered a misidentification and is re-named as pX [70].

Genome replication follows the standard positive-stranded RNA virus model. Capsid assembly, guided by VP0 (containing VP4 and VP2), VP3, and VP1pX, involves cleavage of VP1pX by a cellular protease, leading to the removal of pX from HAV particles [73]. Despite the availability of vaccines [74], specific treatment for HAV infection is lacking, and supportive measures remain the primary approach.

### Role of EVs in HAV replication and transmission

Current knowledge of EVs in HAV replication and transmission extends beyond the traditional non-enveloped transmission route (Fig. 2B). HAV released from cells acquires a protective cloak of host-derived membranes, forming quasi-enveloped “eHAV” [75]. These enveloped viruses, similar to exosomes, maintain infectivity, sensitivity to chloroform extraction, and circulation in the blood. The proposed eHAV biogenesis involves host proteins linked to the ESCRT, specifically VPS4B and ALIX. Membrane hijacking by HAV assists in evading neutralizing antibodies, potentially enhancing virus spread within the liver [75].

Quantitative proteomics analysis of eHAV reveals specific sorting of HAV capsid proteins into vesicles enriched with endolysosomal system components and common exosome-associated proteins like CD9, Dipeptidyl peptidase 4 (DPP4), and ESCRT-III proteins like CHMP2A [76]. Rab5C and Rab7A are implicated as crucial for eHAV biogenesis, although their underlying mechanisms and roles are unclear [76]. Clathrin- and dynamin-dependent endocytosis, facilitated by integrin β1, coordinates the entry of both non-enveloped and eHAV into cells, each employing distinct uncoating mechanisms and release pathways [77]. The viral protein pX, located on the surface of eHAV and absent in non-enveloped virions, plays a crucial role in its biogenesis, potentially through interaction with ALIX via its C-terminal portion on the endosomal membrane [78]. Interaction of HAV capsids with host ESCRT components, particularly ALIX and its paralog Protein Tyrosine Phosphatase Non-Receptor Type 23 (PTPN23), is also reported crucial for eHAV release by promoting the entry of HAV capsids into MVBs [79]. A conserved export signal within the pX extension of VP1 regulates ESCRT-dependent release, resembling late domains of enveloped viruses [79]. Additionally, the NEDD4 family E3 ubiquitin ligase ITCH interacts with pX and plays a crucial role in eHAV release [80]. These findings challenge traditional virus classification, contributing to our understanding of viral pathogenesis and therapeutic targets in HAV-mediated hepatitis.

In the context of eHAV entry, a genome-wide screen identifies components of the ganglioside synthetic pathway, including glucosylceramide synthase, as crucial host factors for cellular entry and the infection process. Specifically, gangliosides, such as disialogangliosides, serve as crucial endolysosome receptors for both non-enveloped and quasi-enveloped HAV virions, undergoing uncoating and accumulating within Lysosomal Associated Membrane Protein 1 (LAMP1)-positive endolysomes, thereby influencing cellular infectivity [81]. Additionally, the phosphatidylserine receptor HAVCR1 and cholesterol transporter NPC1 have been implicated in cargo delivery from exosomes of HAV-infected cells through Clathrin-mediated endocytosis pathway [82]. These receptors interact with MVBs, facilitating membrane fusion between endocytic endosomes and MVBs to delieve cargos into the cytoplasm, revealing an entry pathway independent of envelope glycoproteins [82]. These studies challenge conventional understanding by highlighting envelope-glycoprotein-independent fusion mechanisms shared by exosomes and viruses.

### Future perspectives on HAV and EVs research

Despite the evidence supporting eHAV production through the exosomal pathway and its capability to infect hepatic cells, numerous unanswered questions persist. These inquiries extend to the potential involvement of other EV subtypes, given the notable variations in their production. Moreover, the uncertainty regarding the contribution of an ESCRT-independent exosomal biogenesis pathway, particularly the ceramide-triggered route, adds to the existing gaps in knowledge. Additionally, the involvement of Rab GTPases and their roles in the eHAV vesicular trafficking, biogenesis and release still awaiting futher exploration, albeit Rab5 and Rab7 have been implicated in the biogenesis, but with unclear mechanisms [76].

Further exploration is essential to uncover additional characteristics, such as proteins or lipids, in the composition of the eHAV envelope, surpassing the known elements of CD9, phosphatidylserine, DPP4, as well as the ESCRT-III components like CHMP2A [76, 82]. A specific emphasis should be placed on identifying distinct proteins, including viral proteins in the EVs. Additionally, a comprehensive examination of the entry specificity of eHEV into hepatic cells and the potential regulators involved is warranted, taking into account the expression of HAVCR1 not only in the liver but also in other organs [83]. These areas remain open for future studies, representing unresolved subjects. Addressing these lingering questions can significantly advance our understanding of the mechanisms governing eHAV production and infection.

### Canonical HBV life cycle

HBV, a member of the *Hepadnaviridae* family, possesses a unique relaxed, circular, partially double-stranded DNA structure (rcDNA). Three types of HBV particles are found in infected patients’ serum: Dane particles (42 nm), 22 nm spherical particles, and variable-length filament structures, all enclosing surface antigens (HBsAg). Dane particles, the infectious virions, consist of a host-derived lipid membrane surrounding viral core proteins (HBc) and a nucleocapsid with viral genome DNA. The more abundant 22 nm particles include non-infectious subviral particles (SVPs), while other non-infectious particles lack a viral genome or contain viral RNA [84].

**Fig. 3:**
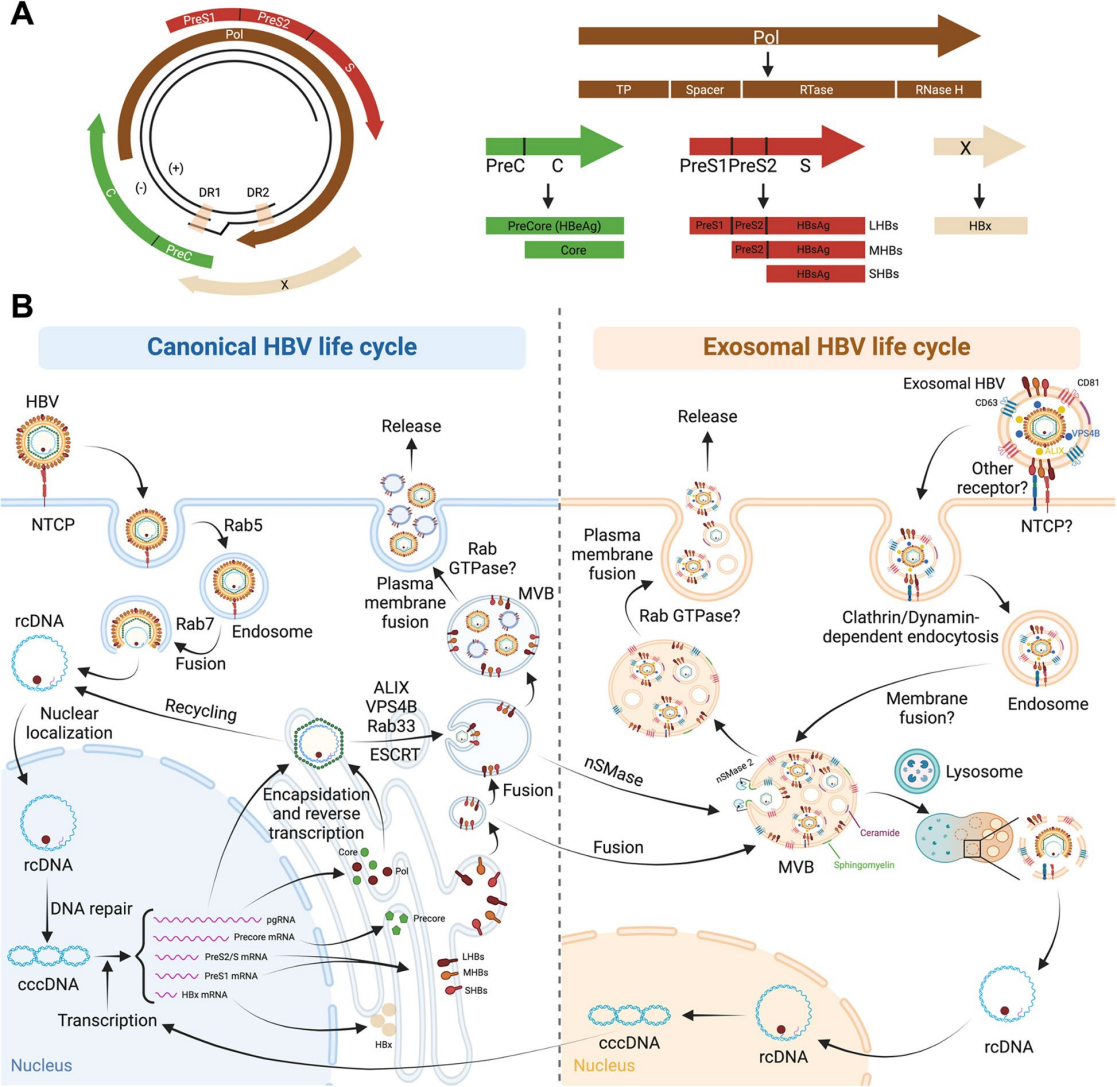
The interplay between canonical and extracellular vesicle-mediated HBV life cycle. **A** The schematic depicts the genome structure of HBV, encompassing four overlapping open reading frames (ORFs) that give rise to crucial components such as HBsAg (LHBs, MHBs, and SHBs), the nucleocapsid containing HBeAg (precore) and core antigens (HBcAg), polymerase (Pol) with reverse transcriptase and DNA polymerase activities, RNase activities, and the potentially hepatocarcinogenic X protein (HBx). **B** Canonically, in HBV-infected cells, the process begins with the binding of the virus to the NTCP receptor, followed by Rab5-medaited endocytosis and Rab7-dependent membrane fusion to facilitate virus uncoating. The relaxed circular HBV DNA (rcDNA) is then transported into the nucleus, where it undergoes conversion into covalently closed circular DNA (cccDNA). The cccDNA serves as a template for the generation of various HBV RNAs, including pregenomic RNA (pgRNA), precore/core mRNA, preS1/preS2/S mRNA, and HBx mRNA, transcribed from different promoters. These transcripts yield protein products such as precore/core, polymerase (Pol), LHBs, MHBs, SHBs, and HBx. The core protein assembles into an icosahedral capsid that encapsulates pgRNA associated with Pol. LHBs, MHBs, and SHBs are translated in the ER lumen and anchored into the ER membrane. These anchored membranes can form vesicles, fusing with endosomes or MVBs, enriching their membranes with LHBs, MHBs, and SHBs. The assembled HBV capsid can enter MVBs via ESCRT proteins like ALIX, VPS4B, and Rab33, overlapping with the ESCRT-dependent exosomal pathway. Host MVB functions are crucial for efficient HBV virion release after fusion with the plasma membrane. Exosomal HBV, a double-layer membrane structure, reportedly contains CD63, CD81, and HBsAg (LHBs, MHBs, and SHBs) on its surface. HBsAg is implicated in binding to the NTCP receptor, potentially facilitating entry via Clathrin/dynamin-mediated endocytosis. Exosomal HBV-containing endosomes may fuse with other endosomes to form MVBs. These MVBs then faces two possible fates: fusion with the lysosome to release the viral genome or re-secretion into the extracellular space as an exosome. The crosstalk between canonical and exosomal HBV replication and transmission involves neutral sphingomyelinase (nSMase), specifically membrane-associated nSMase 2, catalyzing the conversion of sphingomyelin to ceramide, leading to ceramide enrichment on the membrane composition of exosomal HBV. The question mark denotes an unknown or unclear process and molecular mechanism

The HBV genome encodes four overlapping open reading frames (ORFs), generating crucial components such as HBsAg, nucleocapsid with HBeAg and core antigens (HBcAg), polymerase with reverse transcriptase, DNA polymerase, and RNase activities, and the potentially hepatocarcinogenic X protein (HBx) [85, 86] (Fig. 3A).

Currently, HBV entry has been considered to be NTCP receptor dependent, followed by Clathrin- and Dynamin-dependent endocytosis after virions attached [87]. In infected cells, rcDNA converts into covalently closed circular DNA (cccDNA), producing HBV RNAs transcribed from different promoters, yielding protein products like LHBs, MHBs, SHBs, and HBx. The HBc protein forms an icosahedral capsid incorporating 3.5 kb viral pregenomic RNA (pgRNA) associated with Pol. HBe is produced through translation of the 3.5 kb preC mRNA. Pol, the largest HBV protein, has four domains: terminal protein (TP), spacer, reverse transcriptase (RT), and ribonuclease H (RNaseH). The three HBs proteins share a common C-terminal S region, with MHBs carrying an extended preS2 region at the N-terminus. LHBs include a preS1 region at the N-terminus of preS2 and S regions, crucial for receptor binding during virus entry. HBs proteins undergo translation and post-translational modification in the endoplasmic reticulum (ER) lumen before being anchored on the ER membrane, facing the lumen side. ER-derived vesicles containing anchored HBs proteins are subsequently transported to and fused with host MVBs, resulting in MVBs with anchored HBs proteins facing the MVB lumen [88]. Host MVB functions are crucial for efficient budding and release of enveloped HBV virions in an ESCRT component (ALIX, and VPS4B)-dependent manner, overlapping with the biogenic machinery of the ESCRT-dependent exosomal pathway [89, 90]. Additionally, HBe-mediated Rab7 activation and subsequent Rab7-mediated alteration of trafficking to the degradative lysosome reveal a suppressive role of Rab7 in HBV replication and secretion [91]. Additionally, Rab5 has been shown to be important for HBs trafficking from the ER to MVBs [88]. Rab33B is implicated in controlling HBV assembly by regulating HBc levels without affecting viral transcription and inhibiting core/nucleocapsid sorting to envelope-positive intracellular compartments [92].

Anti-HBV therapy is recommended for patients with chronic HBV infection, characterized by active viral replication markers (including HBeAg and HBV-DNA positivity) and evidence of hepatic damage (elevated liver enzymes and active necroinflammatory lesions on liver biopsy). Notably, occult HBV infection (OBI), a specific form of chronic HBV infection, involves replication-competent viral DNA in the liver (with detectable or undetectable HBV-DNA in the serum) among individuals testing negative for HBsAg [93].

### Current insights on EVs in HBV replication and transmission

In addition to the classical HBV life cycle, a seminal study uncovered the intricate interplay between EVs and HBV infection, unveiling novel facets [94] (Fig. 3B). Using a sophisticated HBV infectious culture system with primary human hepatocytes, Sanada et al. demonstrated that CD63 and CD81-positive exosomes, but not those containing CD9, from HBV-infected cells carry HBV-DNA, transmitting this genetic cargo to naive cells through a ceramide-triggered exosome production pathway, independent of the ESCRT pathway, as evidenced by the use of the nSMase2 specific inhibitor GW4869 [94]. These HBV-DNA-transmitting exosomes resist antibody neutralization, adding an antibody-neutralization-resistant dimension to HBV infection [94]. Subsequent research by Yang et al. confirmed this phenomenon in a clinical setting, revealing that exosomes from sera of chronic HBV (CHB) infected patients contain HBV-DNA and proteins, actively transferring HBV to hepatocytes [95]. This study confirmed the presence of CD81 and CD63 in the isolated exosomes, demonstrating detectable HBV-DNA in nature killer (NK) cells after exposure to HBV-positive exosomes. The entry of HBV-positive exosomes into NK cells impairs their functions, suggesting an unexplored route in HBV transmission and the induction of NK-cell dysfunction during CHB infection [95]. Ninomiya et al. subsequently addressed hepatic trafficking pathways used by HBV, revealing that while CD63 is required for HBV particles, it may not be indispensable for HBV-containing exosomes [96]. They showed that CD63 depletion led to intracellular accumulation of LHBs protein and a reduction in the infectivity of released HBV particles, but did not alter the levels of either intracellular or extracellular HBV-DNA, nor pregenomic RNA, establishing CD63 as a marker and essential component in the intricate process of highly infective HBV particle formation and release [96].

Recently, Wu et al. demonstrated that intact virions can be released wrapped in exosomes [97]. Using advanced exosome isolation and characterization techniques, they efficiently separated exosomes from free virions, and limited detergent treatment of exosomes facilitated the stepwise release of intact HBV virions and naked capsids. Contrary to previous studies reporting the absence of HBsAg on the exosome surface [94] and the NTCP-independent entry of HBV-containing exosomes into NK cells [95], Wu et al. showed the presence of intact virions in exosomes with LHBs observed on their surface. Moreover, they demonstrated that the entry of HBV-containing exosomes into cells occurs in an LHBs/NTCP-dependent manner, although uptake of exosomal HBV with low efficiency was also observed in non-permissive cells, unveiling a previously undescribed entry/release pathway for HBV-containing exosomes [97].

### Future perspectives on HBV and EVs research

While shedding light on various facets of EV-mediated HBV transmission, these findings challenge traditional perspectives and offer insights into therapeutic targets and viral pathogenesis in HBV infection [98]. Several questions persist, including uncertainties about the presence of HBsAg on the surface of HBV-containing exosomes and its necessity for exosomal HBV entry. Additionally, the potential dispensability of a specific receptor like NTCP for HBV-containing exosomes requires clarification. Despite the understanding that the canonical HBV life cycle involves the ESCRT pathway and Rab GTPases [89–91], their involvement in exosomal HBV biogenesis and release remains unclear. With advancements in EV subtype isolation approaches, investigating the participation of other EV subtypes in HBV transmission becomes crucial. Lastly, considering the possibility of OBI transmission through EVs, exhibiting HBsAg negativity and HBV-DNA positivity, exploring potential differences in EV composition and the presence of HBV within exosomes and other EV subtypes could provide valuable insights. Remarkably, in the absence of CD63 expression, intracellular HBsAg accumulated without affecting the levels of either intracellular or extracellular HBV-DNA, nor pregenomic RNA, resembling the characteristics of OBI, albeit with a observed reduction in the infectivity of released HBV particles [96]. This observation suggests that CD63 may be implicated in the development of OBI. These aspects remain subjects for further in-depth investigations.

### Canonical HCV life cycle

**Fig. 4:**
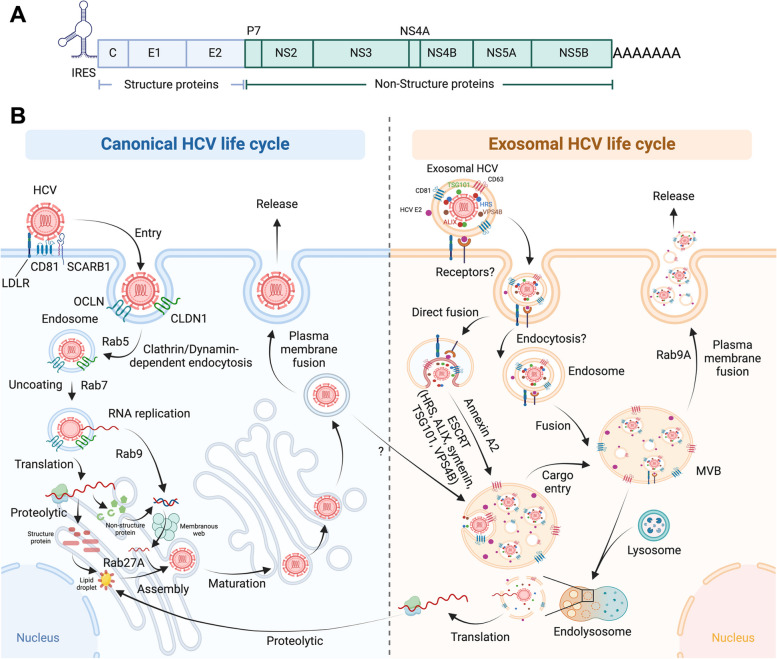
The interconnection of canonical and extracellular vesicle-mediated HCV life cycle. **A** The schematic outlines the genome structure of HCV, encompassing ten viral proteins expressed through a polyprotein via an internal ribosome entry site (IRES). **B** The canonical HCV life cycle initiates with binding and attachment to receptors such as LDLR, CD81, and SCARB1 on cells, facilitating entry into hepatocytes. Following initial attachment, claudin-1 (CLDN1) and Occludin (OCLN) mediate translocation to tight junctions and promote Clathrin/dynamin-mediated endocytosis, with the involvement of Rab5. After Rab7-mediated uncoating, viral genomic RNA is released for translation and RNA replication, facilitated by Rab9. Polyprotein translation begins in the endoplasmic reticulum (ER), where ribosomal subunits bind to HCV-RNA. After proteolysis, mature viral proteins induce the rearrangement of host cell membranes, forming double-membrane vesicles within the membranous web. The NS5B RNA-dependent RNA polymerase catalyzes negative-sense RNA synthesis, producing positive-sense progeny HCV-RNA. Newly synthesized HCV-RNAs are utilized for translation and replication, assembling near cytosolic lipid droplets. Assembly involves fusion with luminal lipid droplets in the ER. Subsequent transportation and maturation occur through the Golgi-mediated very-low-density lipoproteins (VLDLs) pathway before packaging and release. In exosomal HCV, the exosome surface reportedly contains CD81, CD63, and HCV E2 protein, while ESCRT-related proteins such as HRS, TSG101, VPS4B, and ALIX are localized within exosomal HCV. Although the entry process for exosomal HCV remains unclear, it may occur through fusogenic or endocytic pathways. Exosomal HCV can be released into cells either by direct fusion with the plasma membrane or through early endosomes from the endocytic pathway, which may fuse with multivesicular bodies (MVBs). MVBs subsequently face two possible fates: fusion with lysosomes for the release of the viral genome or re-secretion into the extracellular space as an exosome via a Rab9A-dependent route after fusion with the plasma membrane. The crosstalk between canonical and exosomal HCV replication and transmission may involve Annexin A2 and ESCRT machineries like HRS, ALIX, TSG101, VPS4B, and syntenin, although detailed mechanisms remain unclear. The question mark denotes an unknown or unclear process and molecular mechanism

Belonging to the *Flaviviridae* family, HCV is a single-stranded RNA virus transmitted parenterally [99]. Its 9.6 kb RNA genome encodes ten viral proteins, including core (C), E1, E2, p7, NS2, NS3, NS4A, NS4B, NS5A, and NS5B, expressed through a polyprotein via an IRES (Fig. 4A). The HCV life cycle commences with the binding to cells, facilitated by factors such as proteins, lipids, and glycans, resulting in entry into hepatocytes through Clathrin-mediated endocytosis [100–102]. Initial attachment occurs to surface proteoglycans, including Scavenger Receptor Class B Member 1 (SCARB1) and CD81, followed by claudin-1 and Occludin-mediated translocation to tight junctions. Subsequent clathrin-mediated endocytosis and low-pH fusion with endosomal membranes release viral genomic RNA for translation and RNA replication, with regulation by Rab5 and Rab7 thought to govern these early entry processes [103, 104]. Polyprotein translation initiates in the ER when ribosomal subunits bind to HCV-RNA [105].

Viral proteolytic processing within the ER involves cleavage by cellular proteases and the NS2 cysteine protease. NS3, assisted by NS4A, forms a protease complex, resulting in 10 mature HCV proteins [106]. HCV proteins induce rearrangement of host cell membranes, forming double-membrane vesicles in the membranous web. The NS5B RNA-dependent RNA polymerase catalyzes negative-sense RNA synthesis, generating positive-sense progeny HCV-RNA [107]. Interestingly, Rab27A is involved in the control of HCV-RNA replication in a miR-122-dependent manner [108].

Newly synthesized HCV-RNAs are employed for translation and replication, assembling near cytosolic lipid droplets. Core proteins, along with host diacylglycerol acetyltransferase-1 (DGAT1), form the nucleocapsid [109]. Assembly involves fusion with luminal lipid droplets loaded with Apolipoprotein E (ApoE) proteins, creating a high-density HCV precursor. In the Golgi, pre-very-low-density lipoproteins (VLDLs) and the high-density HCV precursor mature before packaging and release. The low-density HCV lipoviral particle, formed by coupling with VLDLs, is transported to the cell surface and released via the cellular VLDL pathway, dependent on Rab9 [110, 111].

Approved direct-acting antivirals (DAAs) can potentially cure most chronic HCV infections, impeding cirrhosis progression if administered early [112]. Achieving a cure reduces liver inflammation, halts fibrosis, and lowers the risk of complications [113]. Occult HCV infection (OCI), characterized by HCV-RNA in hepatocytes or peripheral blood mononuclear cells (PBMCs) without detectable serum HCV-RNA, represents a specific form of chronic HCV infection [114].

### Current understanding of EVs in HCV replication and transmission

The utilization of the EV secretion pathway, specifically employing exosomes for HCV transmission, was proposed in the early 2000s, preceding advancements in other hepatotropic viruses (Fig. 4B). Masciopinto et al. initially suggested that in the absence of CD81, HCV envelope proteins were predominantly confined to the ER [115]. Conversely, in the presence of CD81, these proteins traversed the Golgi, underwent post-translational modifications, and were subsequently located within EVs, specifically exosomes with a diameter of 60–100 nm, larger than HCV particle diameter ranging from 40–80 nm, enriched with CD81. These exosomes circulated, utilizing their fusogenic capabilities to infect cells, even in the presence of neutralizing antibodies. The study also presented clinical evidence from HCV patient sera, supporting the presence of the HCV genome within CD81-positive exosomes, although with a relatively small number of cases.

However, despite this discovery, significant progress in understanding the relationship between EVs and HCV did not occur until 2012—an era characterized by burgeoning advancements. Tamai et al. demonstrated that HCV secretion from host cells necessitates the ESCRT-dependent exosomal pathway, specifically through the HRS-dependent ESCRT-0 pathway, potentially enriched in CD63-positive exosomes [116]. This study also showed that both the HCV core protein and E2 envelope protein were detected in the ILVs of MVBs.

In the same year, Dreux et al. illustrated the entry of HCV-RNA-containing exosomes into non-permissive dendritic cells, indicating the existence of an alternative, canonical HCV receptor dispensable entry route [117]. This study not only affirmed HCV’s reliance on hijacking the ESCRT pathway for exosome secretion but also showcased the utilization of ceramide/nSMase2 pathway, as well as the dependence on Annexin A2, an RNA-binding protein involved in membrane vesicle trafficking, for the secretion of HCV-containing exosomes. These findings suggested that the vesicular sequestration and exosomal export of HCV-RNA may function both as a viral strategy to evade pathogen sensing within infected cells and as a host strategy to induce an unopposed innate response in HCV replication-nonpermissive cells.

A subsequent confirming study by Ramakrishnaiah et al. demonstrated that exosomes isolated from HCV-infected hepatoma cells could exhibit the presence of the HCV core protein and transmit HCV to naive cells [118]. Even with subgenomic replicons lacking structural viral proteins, exosome-mediated transmission of HCV-RNA was observed, highlighting the potential of HCV transmission by exosomes resistant to neutralizing antibodies as an immune evasion mechanism. Although Masciopinto et al. had demonstrated the presence of the HCV genome within patient sera-derived exosomes as early as the 2000s [115], the infectivity of these patient sera-derived HCV-containing exosomes remained unknown until Bukong et al. demonstrated that the isolated HCV-containing exosomes were infectious and transmissible, thereby confirming the existence of transmissible HCV-containing exosomes in clinical settings using sera from chronic HCV-infected patients [119].

Not only has the ESCRT protein HRS been reported to play a crucial role in the entry into MVBs and the biogenesis of HCV component-containing exosomes, as demonstrated in previous studies [116, 117], but also the syntenin-ALIX complex has been implicated in mediating entry into MVBs, possibly in conjunction with TSG101 [120]. This study revealed that increased syntenin expression led to a reduction in intracellular HCV E2 protein abundance while simultaneously increasing the secretion of E2-coated exosomes. However, these exosomes were found to lack infectivity. Interestingly, the presence of the nSMase2 specific inhibitor GW4869 significantly reduced the production of E2-coated exosomes. These findings suggest that although HCV E2 may assist in the biogenesis of HCV component-containing exosomes, this specific type of exosome is not responsible for HCV transmission. The biogenesis pathways may involve both the ESCRT/syntenin-ALIX complex and the ceramide/nSMase pathways. Similarly, another study also demonstrated that the HCV replication intermediate, specifically the innate immunity inducible double stranded HCV-RNA, could be packaged within exosomes, generated through ceramide/nSMase2 and Rab27-dependent pathway, thereby representing a mechanism to avoid excessive activation of cell intrinsic innate immunity [121].

### Future perspectives on HCV and EVs research

While the exploration of the intricate interplay between EVs, specifically exosomes, and HCV transmission and replication appears comprehensive, several unanswered questions persist. These uncertainties include investigations into whether other EV subtypes play a role in HCV transmission and replication, the potential specificities in the exosomal surface proteins of HCV-containing EVs crucial for the entry of HCV into non-permissive cells like PBMC, besides the E2 protein, as E2-coated exosomes lack infectivity [120]. Additionally, although Rab27A has been implicated in exosomal HCV release, the involvement of Rab GTPases and other molecules in the entry or trafficking of exosomal HCV also remains unclear.

Furthermore, despite achieving a virological cure following DAA treatments, a subset of patients with fibrosis and cirrhosis remains susceptible to liver disease progression or complications [122]. The connection of this phenomenon to OCI and whether it is mediated through EVs remains unknown, necessitating further comprehensive investigations.

Notably, the expression of Acireductone Dioxygenase 1 (ADI1) has been demonstrated to facilitate HCV infection in non-permissive cells when co-expressing CD81 [123, 124]. This phenomenon may be attributed to the potential function of CD81 expression as a viral receptor and the regulation of the lipid raft marker CAV1 level by ADI1 [125], thereby facilitating HCV entry and transmission, although the underlying mechanisms remain unclear.

### Canonical HDV life cycle

HDV, belonging to the *Deltaviridae* family, is a defective RNA-containing passenger virus with a ~ 1.7 kb RNA genome that encodes a single antigen (HDAg) crucial for replication and virion assembly. Operating as a satellite virus, HDV relies on the helper functions of HBV, including the provision of the HBsAg coat, for virion assembly and penetration into hepatocytes [126]. HDV-RNA can be amplified through the expansion (regeneration) of hepatocytes in the absence of HBV [127]. The global prevalence of HDV correlates with that of HBV, and declines due to decreased HBV infection rates after universal hepatitis B vaccination [128]. HDV infection can occur through simultaneous infection with both HBV and HDV (co-infection) or acquiring HDV after an initial HBV infection (super-infection). The combination of HBV and HDV, particularly for specific sub-genotypes, represents the most severe form of chronic viral hepatitis, characterized by a faster progression toward liver-related death and HCC [129].

The life cycle of HDV begins with binding to heparan sulfate proteoglycans (HSPGs) on the hepatocyte membrane, an essential step for the specific interaction of the preS1 domain of the HBV LHBs with the hepatocyte-specific receptor NTCP [130]. Following membrane fusion by an as-yet-unknown mechanism, the HDV genome-containing ribonucleoprotein (RNP) complex is transported to the nuclear pore complex and released into the nucleoplasm. Replication occurs via a double rolling circle amplification mechanism, generating linear multimeric anti-genomic (-) and genomic ( +) RNAs cleaved to monomers by two intrinsic ribozymes. Monomers self-ligate to form sense and antisense single-stranded RNA circles [131]. Genomic RNA serves as the template for mRNAs encoding the two forms of HDAg. During replication, host adenosine deaminase acting on RNA 1 (ADAR1) edits anti-genomic RNA, introducing an A/G mutation in the amber stop codon of the SHDAg ORF, resulting in a Trp codon [132]. Consequently, a second mRNA is produced, coding for the elongated LHDAg with a C-terminal ORF extension of 19 or 20 (genotype-dependent) amino acids. LHDAg becomes prenylated by cellular farnesyl transferase at a conserved C-terminal Cys residue within the extension. Progeny HDV-RNAs assemble to RNPs containing SHDAg, as well as prenylated and non-prenylated LHDAg [133]. These RNPs bud into the host ER where HBs proteins localize. Prenylated Cys-residues of the LHDAg interact with the cytoplasmic domain of SHBs for the acquisition of the HBV envelope, leading to virion release.

Despite HDV infection being preventable through HBV immunization, treatment success rates for chronic infection remain low [134]. Pegylated interferon-alpha is generally recommended for HDV infection despite a relatively low response rate, as it is associated with a reduced likelihood of disease progression [130]. However, significant side effects are linked to this therapy, making it unsuitable for patients with decompensated cirrhosis, active psychiatric conditions, or autoimmune diseases [135]. Bulevirtide, Lonafarnib, and REP2139 thus emerge as promising new treatments for HDV [130]. Nevertheless, efforts to alleviate the global burden of CHB and develop safe, effective, and affordable medicines for HDV, especially for those most in need, remain crucial.

### Current understanding of EVs in the replication and transmission of HDV

**Fig. 5:**
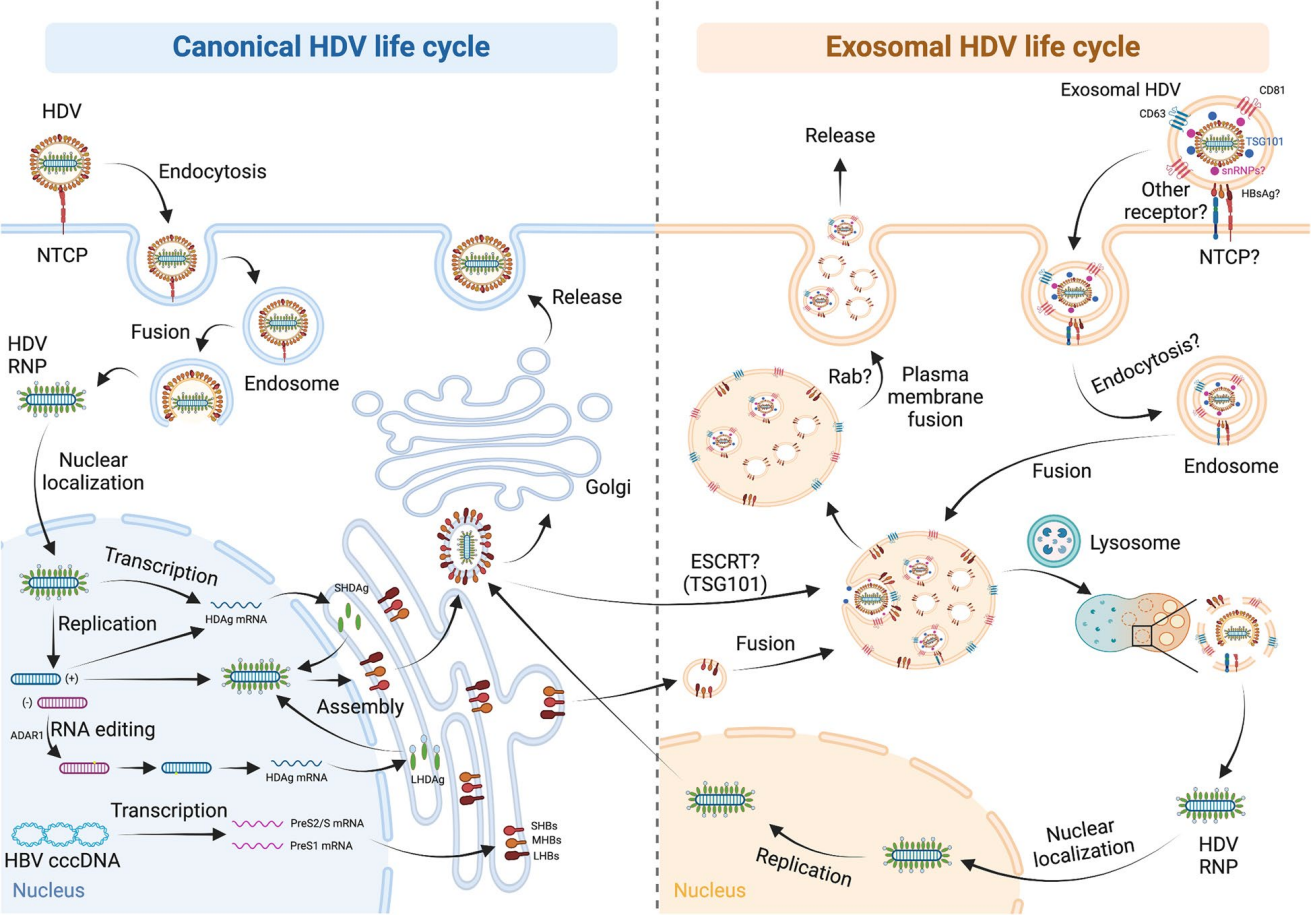
The interplay between canonical and extracellular vesicle-mediated HDV life cycle. The canonical life cycle of HDV initiates with the binding of the virus to the hepatocyte membrane receptor NTCP through the HBV surface proteins (HBsAg). Following membrane fusion, facilitated by an as-yet-unknown mechanism likely similar to that of HBV, the ribonucleoprotein (RNP) complex containing the HDV genome is released and transported to the nucleus. Genomic ( +) RNA serves as the template for mRNAs encoding HDAg, specifically SHDAg. Genome replication occurs through a double rolling circle amplification mechanism, generating anti-genomic (-) and genomic ( +) RNAs. During replication, host adenosine deaminase acting on RNA 1 (ADAR1) edits anti-genomic RNA, generating a second mRNA coding for the elongated LHDAg. Progeny HDV-RNAs assemble into RNPs containing SHDAg and LHDAg in the nucleus and then bud into the host endoplasmic reticulum (ER) where HBsAg proteins localize. LHDAg interacts with the HBsAg proteins for the acquisition of the HBV envelope, leading to virion release. Details regarding the exosomal HDV life cycle remain unclear. However, reports indicate the presence of HDV-RNA in exosomes enriched with CD63 and CD81 surface markers, as well as the ESCRT machinery component TSG101. Although the proposed interconnection between the canonical and exosomal HDV life cycle is depicted, further investigations are warranted for solidification. The question mark denotes an unknown or unclear process and molecular mechanism

The current understanding of the relationship between HDV and the utilization of EVs remains limited (Fig. 5). In 2020, Jung et al. demonstrated that EVs derived from HDV-infected cells could trigger an inflammatory response in non-permissive cells like PBMCs and macrophages [136], suggesting potential transmissibility through hijacking the EVs pathway. However, details regarding the presence of the HDV genome, the contents of these EVs, as well as the isolated EV subtypes remain unclear.

Building on this, in 2021, Cunha et al. made progress in elucidating the involvement of EVs in HDV transmission [137]. They established an HDV-expressing cell line, isolated EVs from the culture media, examined the presence of HDV-RNA in the EVs, and identified enriched CD63, TSG101, and CD81 EV markers in HDV-containing EVs. Intriguingly, they also revealed the enrichment of small nuclear ribonucleoproteins (snRNPs) and RNA-binding proteins in HDV-containing EVs, possibly due to the nature of the RNA genome. Despite these findings, the transmission of HDV through EVs remains inconclusive due to methodological limitations, such as the lack of direct visualization under electron microscopy and concerns about the EV isolation process. Consequently, this research area is less explored compared to other hepatotropic viruses and awaits further comprehensive investigations.

On the other hand, interestingly, Yao et al. introduced an innovative approach to inducing an immune response against HDV replication in vivo by utilizing engineered MVs loaded with ubiquitinated HDAg [138], thereby providing a promising avenue for future therapeutic strategies targeting HDV.

### Canonical HEV life cycle

**Fig. 6:**
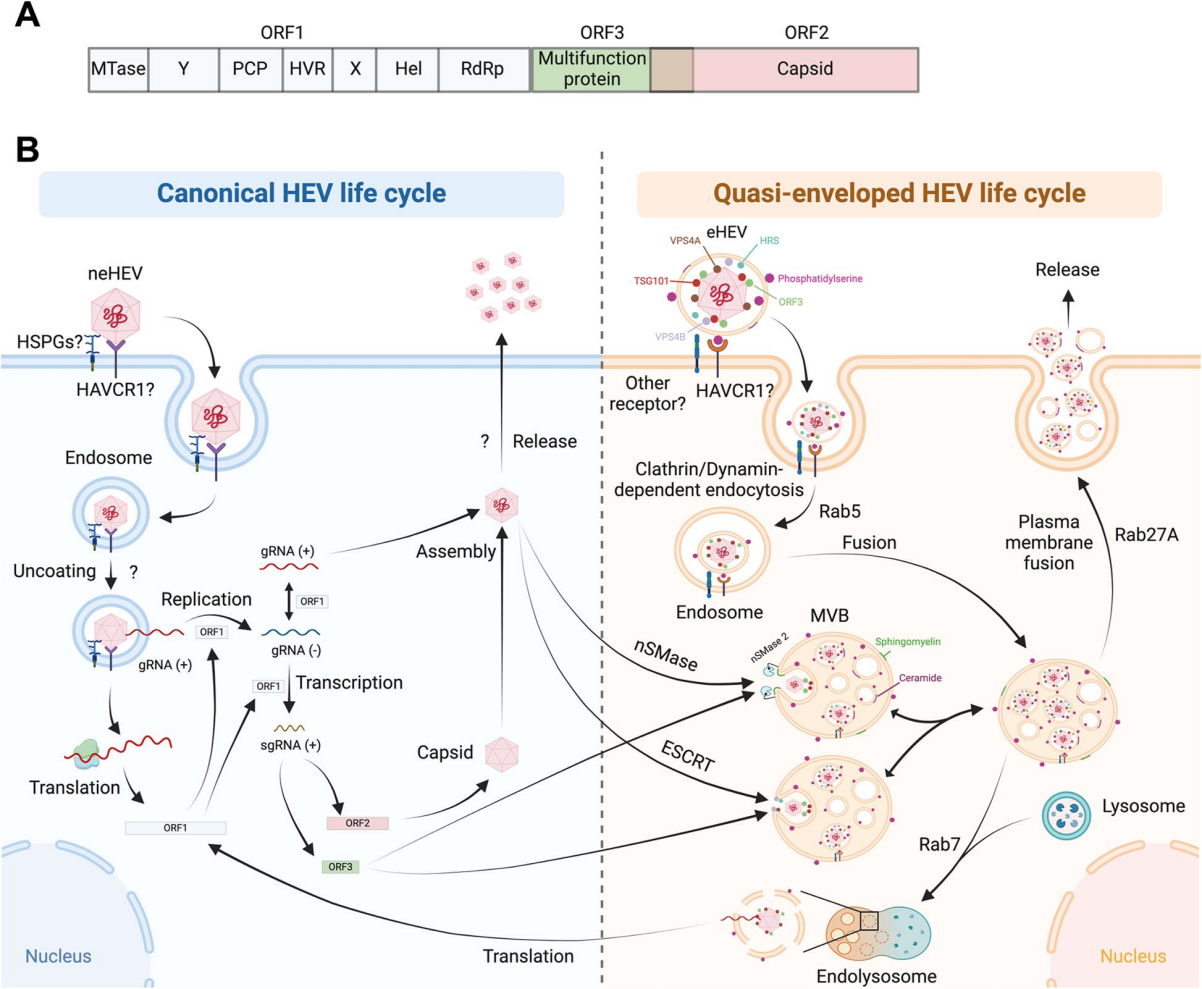
The crosstalk between canonical and extracellular vesicle-mediated HEV life cycle. **A** The schematic illustrates the genome structure of HEV, featuring three open reading frames (ORFs): one for the viral replicase (ORF1), another for the capsid (ORF2), and a third (ORF3) encoding a small protein involved in virion secretion. **B** The HEV life cycle initiates with the initial contact between HEV and host cells, mediated by interactions with receptors such as HSPGs and HAVCR1, which are not fully characterized. After endocytosis and uncoating, the viral genome is released into the cytoplasm, where the host translational machinery promotes translation and generates the ORF1 replicase. The ORF1 replicase facilitates viral RNA replication and generates a negative-strand RNA intermediate serving as a template for two mRNAs, one for ORF2 and another for ORF3. ORF2 is utilized for capsidation, leading to subsequent virion assembly and release, although the mechanism of non-enveloped HEV (neHEV) release is currently unknown. In exosomal HEV (or quasi-enveloped HEV, eHEV), the exosome surface reportedly contains phosphatidylserine but lacks known surface protein markers. However, ESCRT-related proteins such as TSG101, HRS, VPS4A, and VPS4B are localized within eHEV. Additionally, HEV ORF3 is also found within eHEV. The entry of eHEV is reported to involve Clathrin/Dynamin-dependent endocytosis, facilitated by Rab5. The endosome containing eHEV enters multivesicular bodies (MVBs), perhaps through membrane fusion, and then faces two possible fates: fusion with the lysosome to release the viral genome or re-secretion into the extracellular space as an exosome, facilitated by Rab27A. The crosstalk between canonical and eHEV replication and transmission involves ORF3-mediated entry of the MVBs thorugh direct interaction with ESCRT machinery such as TSG101 and VPS4B. Additionally, it is reported that neutral sphingomyelinase 2 (nSMase 2) contributes an important role in regulating eHEV biogenesis, although the detailed mechanisms remain unclear. The question mark denotes an unknown or unclear process and molecular mechanism

HEV is a small, non-enveloped, icosahedral virus with a diameter ranging from 27 to 34 nm, belonging to *Hepeviridae* family. It carries a 7.2 kb single-stranded, positive-sense RNA genome featuring an m7G cap at its 5′ end and a poly-A tail at its 3′ end [139]. The HEV genome encompasses three ORFs: one for the viral replicase (ORF1), another for the capsid (ORF2), and a third for a small protein involved in virion secretion (ORF3), potentially through its ion channel activity [140] (Fig. 6A). The HEV life cycle commences with the initial contact between HEV and host cells, mediated by interactions with receptors that are not fully characterized. These receptors include HSPGs, Asialoglycoprotein Receptor 1/2 (ASGPR1/2), Integrin α3 (ITGA3), ATP Synthase Subunit 5β (ATP5B), Glucose-Regulated Protein 78 (GRP78), Heat Shock Cognate Protein 70 (HSC70), T Cell Immunoglobulin Mucin Domain 1 (TIM-1 or HAVCR1), and Epithelial Growth Factor Receptor (EGFR) [139, 141].

After endocytosis, the viral genome is released into the cytoplasm, where the host translational machinery generates the ORF1 replicase, facilitating viral RNA replication (Fig. 6B). During this stage, two RNA species emerge from a negative-strand RNA intermediate: a full-length genomic RNA and a subgenomic RNA of 2.2 kb. The translation of the subgenomic RNA results in the synthesis of the ORF2 and ORF3 proteins. Subsequent phases of the HEV life cycle involve viral assembly and the release of newly produced virions. It is worth noting that, similar to HAV, HEV appears in two forms: the naked form, predominantly found in bile and feces, and the “quasi-enveloped” virion (eHEV), primarily present in blood [139].

### Current understanding of EVs in the replication and transmission of HEV

The correlation between the presence of eHEV and the utilization of the EV pathway, specifically the exosomal pathway, was demonstrated as early as in 2000s to 2010s, scientists found that although HEV particles present in faeces and bile are non-enveloped, those in circulating blood and culture supernatant have been found to be covered with a cellular membrane, similar to enveloped viruses, and suggest that the important viral factor ORF3 responsible for release, at least its C-terminal portion, is present on the surface of eHEV [142, 143] (Fig. 6B). Virions of eHEV obtained from patient sera can be transmitted in both permissive and non-permissive cell lines, regardless of the coexistence of HEV antibodies [144]. This type of HEV was found to utilize the ESCRT and MVB pathways to release eHEV particles, as evidenced by silencing or loss-of-function of TSG101, VPS4A and VPS4B [145]. Additionally, it was subsequently found that a significant reduction in extracellular eHEV upon treatment with nSMase2 inhibitor GW4869 for the ceramide-triggered exosomal pathway and HRS silencing for ESCRT-dependent exosome secretion. Additionally, Rab27A was shown to be crucial in the release of eHEV. This finding confirmed that the production of eHEV can utilize both ESCRT-dependent and further suggested the utilization of independent exosomal pathways [146]. Subsequent studies addressed the transmissibility of exosome-mediated eHEV secretion and concurrently explored the membrane lipid compositions utilized by eHEV, the endosomal trafficking machinery like Rab5 and Rab7 required for cellular entry, and the immune-evading potential of eHEV [147–149].

While the quasi-envelope of eHEV serves as an elegant strategy for evading antibody-mediated immune responses [147, 149], it also introduces additional steps during cellular entry before genome uncoating. Initially considered to be purely mediated through clathrin- and dynamin-dependent endocytosis pathways with specificity to hepatic cells [148, 150], the entry of eHEV was later elucidated through demonstrating that the phosphatidylserine-enriched membrane of eHEV is crucial for TIM-1 (HAVCR1)-mediated cell entry [151]. Moreover, a recent study revealed new aspects of the HEV life cycle, suggesting that replication and release could be coupled at the endosomal interface, as evidenced by the localization of HEV replicase within MVB and released exosomes [152].

### Future perspectives on HEV and EVs research

Despite these existing pieces of evidence supporting the production of eHEV through the exosomal pathway and its ability to infect hepatocytes, several unanswered questions persist. These include, but not limited to, the same question for other hepatotropic viruses, whether other EV subtypes are involved in the production of eHEV, and whether additional eHEV envelope compositions exist beyond lipid compositions phosphatidylserine, sphingomyelin, ceramides, cholesterol, particularly in terms of host proteins like tetraspanins. Moreover, additional intracellular vesicular machinery like other ESCRT components and Rab GTPases promote eHEV biogenesis and release in cooperation with viral proteins such as ORF2 or ORF3. Additionally, the entry specificity of eHEV into liver cells and any potential regulators, considering TIM-1 (HAVCR-1) expression not only in the liver but also in other organs, remain subjects for future studies [83]. Addressing these issues will contribute to a more comprehensive understanding of the mechanisms underlying eHEV production and infection.

## Conclusion remarks

Delving into the realm of EVs and their role in hepatotropic virus transmission has unveiled diverse facets of viral replication, transmission, and pathogenesis. Particularly noteworthy is the prominence of exosomes as central carriers for hepatotropic viruses, assuming essential functions in viral replication, transmission, and immune evasion. Table 1 summarizes the current understanding of the biogenesis of exosomal hepatotropic viruses and their utilization of cellular vesicle machineries.

**Table 1:** Summary of current understanding in the host vesicular machinery utilized by exosomal hepatotropic viruses

	eHAV	eHBV	eHCV	eHDV	eHEV
Receptor	HAVCR1 (TIM-1), NPC1, and disialogangliosides [81, 82]	NTCP [97]	Unknown or no need (directly fusogenic) [115, 117]	Unknown	HAVCR1 (TIM-1) [151]
Protein marker	Phosphatidylserine, CD9, CHMP2A, and DPP4 [76, 82]	CD63, CD81 and LHBs [94–97]	CD81, CD63, TSG101, VPS4B, CHAMP4B, and HCV E2 protein [115–117, 120]	CD63, TSG101, and CD81 [137]	Phosphatidylserine, sphingomyelin, ceramides, cholesterol, TSG101, HRS, VPS4A, VPS4B, HEV ORF3 [142, 146, 147, 151]
Clathrin- and dynamin-dependent endocytosis	Yes [77]	Yes [87]	Employed by canonical HCV entry, but unknown for eHCV	Unknown	Yes [148, 150]
ESCRT utilization	VPS4B, ALIX, CHMP2A and PTPN23 [75, 79]	ALIX, and VPS4B used by canonical HBV replication [89, 90]	HRS, syntenin, ALIX, TSG101, VPS4B, CHAMP4B [116, 120]	TSG101 [137]	HRS, TSG101, VPS4A, VPS4B [146]
nSMase/ceramide utilization	Unknown	Yes [94, 97]	Yes [117, 121]	Unknown	Yes [146]
Rab GTPase utilization	Rab5C and Rab7A regulate eHAV biogenesis and secretion [76]	Rab5, Rab7, and Rab33B used by canonical HBV replication [88, 91, 92]	Rab5, Rab7, Rab9, and Rab27A employed by both canonical HCV replication and eHCV biogenesis [103, 104, 108, 111, 121]	Unknown	Rab27A for release [146], and Rab5 and Rab7 for entry [148]
Viral factor required for exosomal virus	pX [78]	Unknown	HCV E2 protein [120]	Unknown	HEV ORF2 and ORF3 [146]
Clinical evidence	Yes [75]	Yes [95]	Yes [115, 119, 120]	Unknown	Yes [144]

*eHAV* exosomal HAV or quasi-enveloped HAV, *eHBV* exosomal HBV, *eHCV* exosomal HCV, *eHDV* exosomal HDV, *eHEV* exosomal HEV or quasi-enveloped HEV

Despite notable progress in comprehending EV-mediated viral transmission, numerous queries persist in this field. Unexplored territories include the potential participation of other EV subtypes, given that earlier methodologies may struggle to effectively distinguish small ectosomes like MVs from exosomes, and their biogenesis pathways may diverge. Furthermore, uncertainties endure regarding the surface proteins of virus-containing EVs, the exploitation of ESCRT-independent pathways (though demonstrated in certain viruses), and the role of EV-mediated transmission in occult infections, especially OBI and OCI. It is imperative that future research addresses these knowledge gaps to propel our understanding of the intricate mechanisms governing EV-mediated viral transmission, thereby paving the way for innovative therapeutic strategies. In essence, the exploration of EVs in the context of hepatotropic viruses signifies a dynamic and evolving field with considerable implications for public health and clinical interventions.

## Data Availability

Not applicable.

## Abbreviations

EV: Extracellular vesicle
MVB: Multivesicular body
MV: Macrovesicle
ApoBD: Apoptotic body
EM: Electron microscopy
NTA: Nanoparticle tracking analysis
ILV: Intraluminal vesicle
ESCRT: Endosomal sorting complexes required for transport
nSMase: Neutral sphingomyelinase
HIV-1: Human immunodeficiency virus 1
CAV1: Caveolin-1
HCC: Hepatocellular carcinoma
HAV: Hepatitis A virus
HBV: Hepatitis B virus
HCV: Hepatitis C virus
HDV: Hepatitis D virus
HEV: Hepatitis E virus
NTCP: Sodium taurocholate cotransporting polypeptide
HAVCR1: HAV cellular receptor 1
IRES: Internal ribosome entrance site
eIF4G: Eukaryotic initiation factor 4G
eHAV: Quasi-enveloped HAV
HBV rcDNA: HBV relaxed circular DNA
HBsAg: HBV surface antigens
HBc: HBV core proteins
HBcAg: HBV core antigens
SVP: Subviral particle
ORF: Open reading frame
HBx: HBV X protein
cccDNA: Covalently closed circular DNA
pgRNA: Pregenomic RNA
TP: Terminal protein
RT: Reverse transcriptase
RNaseH: Ribonuclease H
HBeAg: HBV e antigen
OBI: Occult HBV infection
CHB: Chronic HBV infection
NK cells: Nature killer cells
SCARB1: Scavenger Receptor Class B Member 1
ER: Endoplasmic reticulum
DGAT1: Diacylglycerol acetyltransferase-1
VLDL: Very-low-density lipoprotein
DAAs: Direct-acting antivirals
OCI: Occult HCV infection
PBMCs: Peripheral blood mononuclear cells
ADI1: Acireductone Dioxygenase 1
HDAg: HDV antigen
HSPGs: Heparan sulfate proteoglycans
RNP: Ribonucleoprotein
ADAR1: Adenosine deaminase acting on RNA 1
snRNPs: Small nuclear ribonucleoproteins
ASGPR1/2: Asialoglycoprotein Receptor 1/2
ITGA3: Integrin α3
ATP5B: ATP Synthase Subunit 5β
GRP78: Glucose-Regulated Protein 78
HSC70: Heat Shock Cognate Protein 70
TIM-1: T Cell Immunoglobulin Mucin Domain 1
EGFR: Epithelial Growth Factor Receptor
eHEV: Quasi-enveloped HEV
